# Recurrent primary hepatic VIPoma treated with a combination of surgical resection and loco-regional therapy

**DOI:** 10.2144/fsoa-2022-0046

**Published:** 2023-03-09

**Authors:** Wafa Dahmani, Sahar Nasr, Kais Maalel, Yasser Becheikh, Ahmed Tibaoui, Zainab Lajmi, Sihem Hmissa, Hanene Jaziri, Nour Elleuch, Aida Ben Slama, Wafa Ben Ameur, Mahdi Ksiaa, Ali Jmaa

**Affiliations:** 1 Gastroeneterology Department, Sahloul University Hospital, 4052, Tunisia; 2 Radiology Department, Sahloul University Hospital, 4052, Tunisia; 3 Pathology Department, Sahloul University Hospital, 4052, Tunisia

**Keywords:** hepatic resection, neuroendocrine tumor, transarterial chemoembolization, Vipoma

## Abstract

Vasoactive intestinal peptide (VIP) secreting tumors (VIPomas) are insidious functional neuroendocrine tumors originating mainly from pancreatic islet cells. Hepatic localization is considered exceedingly rare as only few cases have been reported in the literature. Diagnostic and therapeutic management of this tumor is still not clearly codified and therefore represents a real challenge for clinicians. Herein we report a unique case of a primary hepatic VIPoma recurrence in a female patient 22 years after curative resection. The patient had two sessions of transarterial chemoembolization. Complete symptomatic improvement was achieved since the first day after the first session. This case highlights that long-term follow-up for patients with hepatic VIPoma is mandatory as recurrence could occur several years after curative surgical treatment.

## Plain language summary

VIPomas are rare tumors secreting a hormone that is vasoactive in the intestine causing severe diarrhea. The majority of VIPomas arise within the pancreas. The hepatic localization is extremely rare. We report a case of a very late recurrence of a primary hepatic VIPoma surgically treated 20 years ago. The case was managed with therapeutic radiology by blocking the blood supply to the tumors after administering anticancer drugs in the vessels near them.

Vasoactive intestinal peptide (VIP) secreting tumor (VIPoma) is a rare and insidious functional neuroendocrine tumor (NET) first described by Verner and Morrison in 1958 [1]. VIPoma annual incidence in the general population is estimated to be 1/10,000,000 individuals. VIP overproduction results in profuse watery diarrhea, also known as “pancreatic cholera”, hypokalemia, achlorhydria and in severe cases dehydration and functional renal insufficiency [1]. Although VIPomas often originate from the pancreatic islet cells, cases of extrapancreatic VIPomas have also been described, among which hepatic localization is considered one of the rarest sites [2].

Due to their extreme rarity, no management guidelines of these tumors have been established and both diagnosis and treatment of VIPomas remain challenging. Prognosis is highly variable and is mainly dependent on metastatic extension, tumor growth speed and differentiation. Herein we report a unique case of recurrence of a primary hepatic VIPoma in a female patient occurring more than two decades after initial curative resection.

## Case report

A 58-year-old woman with a history of newly diagnosed diabetes mellitus and primary hepatic VIPoma treated with right liver hepatectomy in 1998 presented in September 2020 to the emergency department with a five-month history of multiple watery, non-bloody diarrhea of up to 20 loose bowel movements per day and a weight loss of 10 kgs. The patient denied nausea, vomiting or abdominal pain. The physical examination revealed no signs of dehydration. The laboratory work-up revealed a potassium level of 1.9 mmol/l, anicteric cholestasis and a normal level of albumin and hemoglobin. The clinical picture was very similar to the one she presented with 22 years ago when the hepatic VIPoma was diagnosed. A recurrence of VIPoma was immediately suspected. The plasma VIP level was 451 pmol/l (upper limit 40 pmol), the chromogranin A level was within normal range.

**Figure 1.**
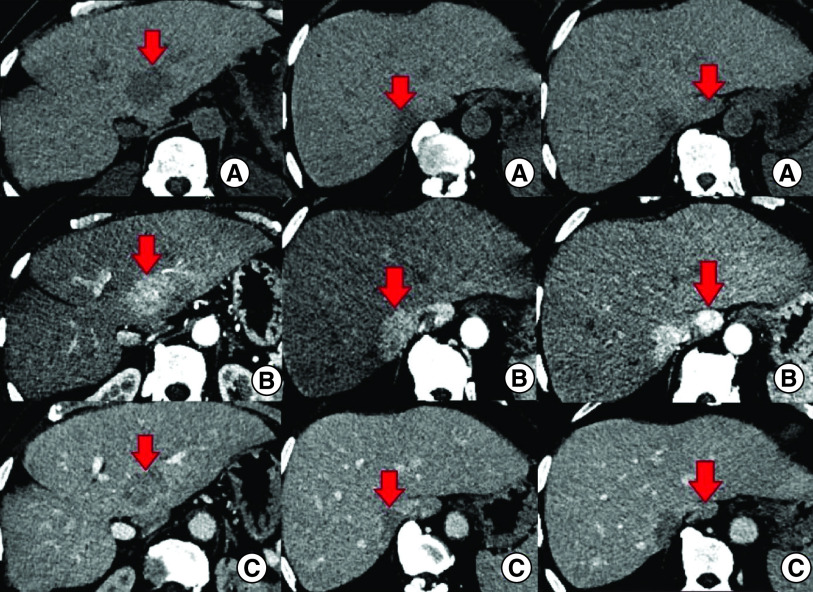
Abdominal CT – axial sections showing the hepatic lesions (red arrows). **(A)** The lesions are spontaneously hypodense. **(B)** After injection of contrast product, the lesions are intensely enhancing in the arterial phase. **(C)** Contrast product wash out in the portal phase.

**Figure 2.**
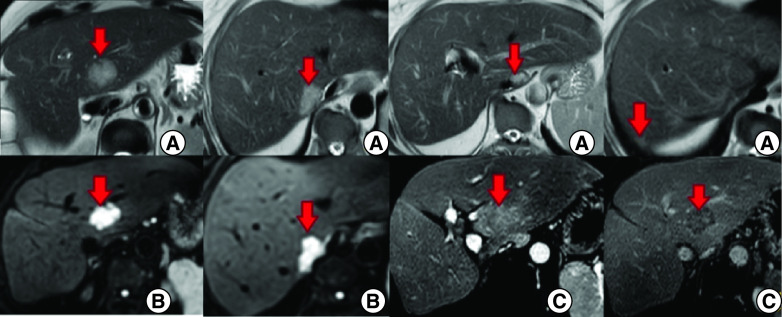
Hepatic MRI – axial sections. **(A & B)** Hepatic lesions (red arrows) in hypersignal T2 and diffusion respectively. **(C)** Post-contrast images show enhancement in the arterial phase with a wash out in the portal and late phases.

**Figure 3.**
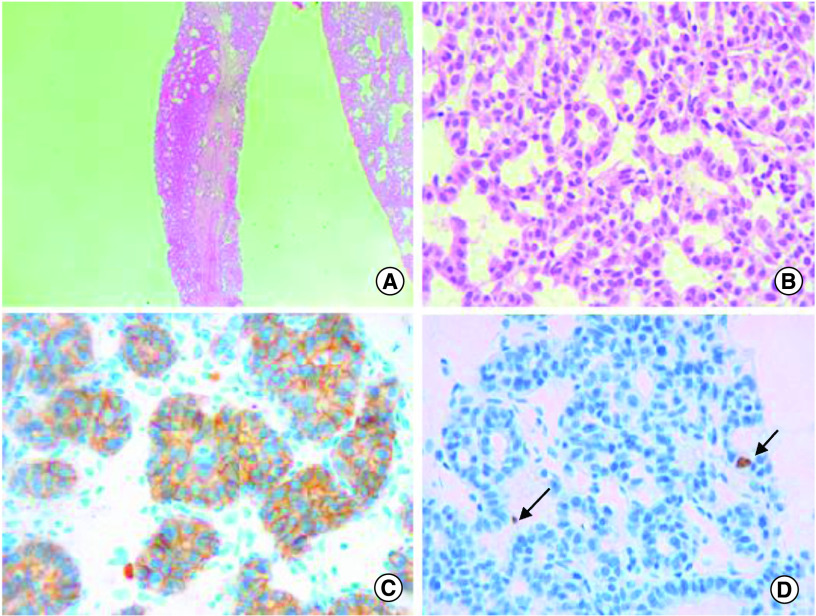
Histological features of the hepatic VIPoma. **(A)** Glandular architecture of the tumor (Hematoxylin-eosin x100). **(B)** Tumor cells are monotonous and display round nuclei and moderate amounts of eosinophilic cytoplasm. Nuclei show a characteristic stippled (“salt and pepper”) chromatin pattern with inconspicuous nucleoli (Hematoxylin-eosin x400). **(C)** The tumor is diffusely and strongly positive for CD56 immunostaining (×400). **(D)** The proliferation marker Ki-67 highlights 2% of the neoplastic cells (arrow), consistent with a low proliferation index (×400).

**Figure 4.**
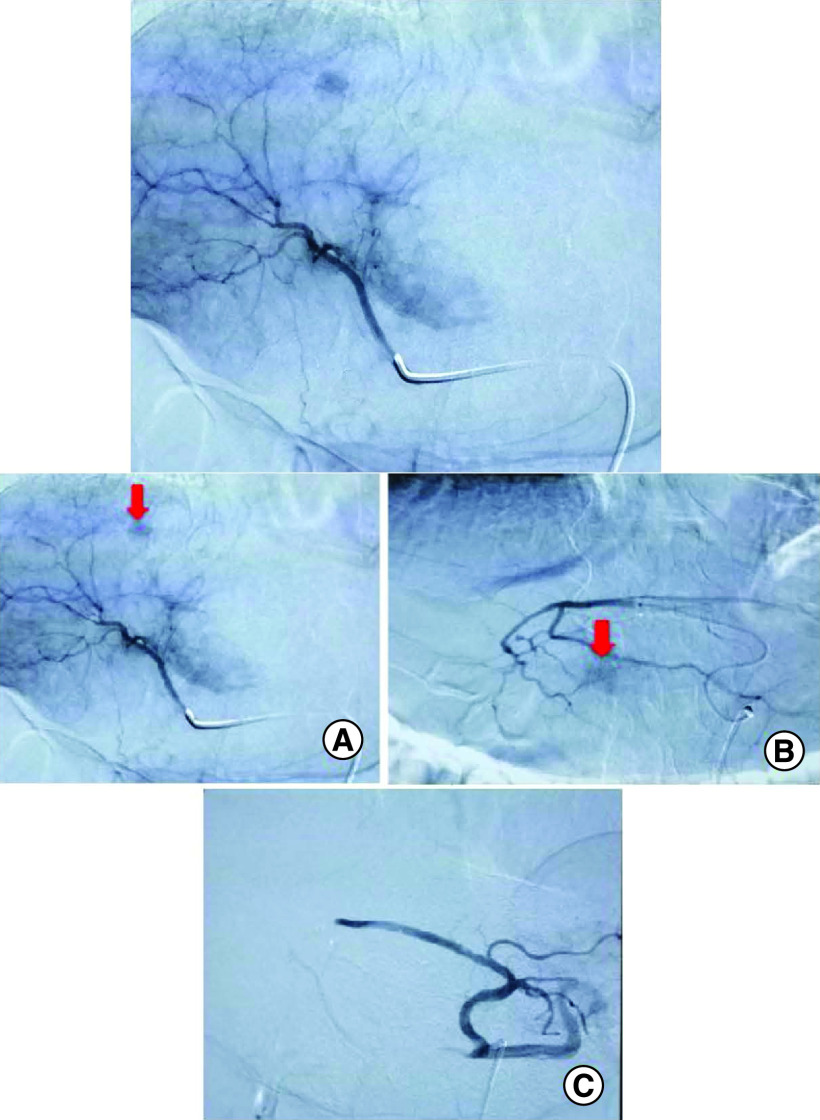
Hepatic arteriogram with selective catheterization of the right hepatic artery from the femoral artery. **(A & B)** A progressive blush of both lesions (red arrows) following the injection of contrast media. **(C)** Angiographic control showing total disappearance of the tumoral blush.

**Figure 5.**
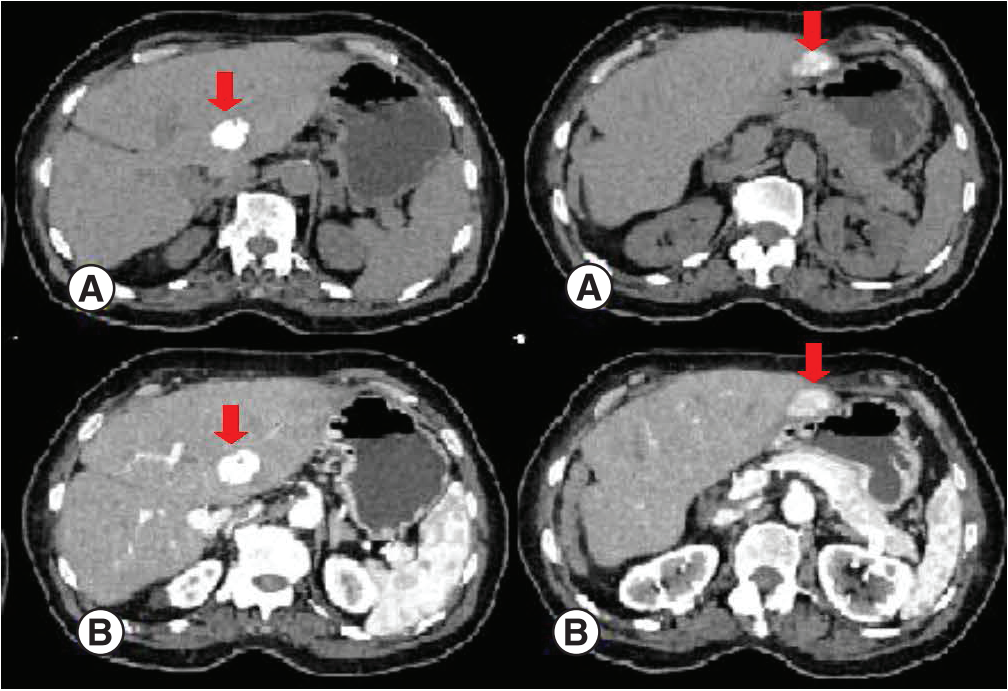
Follow-up abdominal CT taken one month after the second session of transarterial chemoembolization. The CT showed a dense and homogeneous lipiodol deposition in the tumor on precontrast images (red arrow in **[A]**), without persistent arterial phase hyperenhasement (red arrow in **[B]**)**.**

An abdominal computed tomography (CT) scan was performed revealing four hepatic lesions of segments I, III, IV and the sectional slice. The lesions were spontaneously hypodense, intensely enhancing after injection of contrast product in the arterial phase with a wash out in the portal phase (Figure 1). The lesions were hyperintense on T2- and diffusion weighted MRI, enhancing in the arterial phase with a wash out in the portal and late phases (Figure 2). Octreotide scan failed to visualize any hepatic lesion. Ultrasound-guided liver biopsy of the lesions was performed revealing a tumor cell proliferation with glandular architecture. The tumor cells were small, round and monotonous with “salt and pepper” nuclear chromatin. Immunohistochemical staining revealed that tumor cells were diffusely positive for neuroendocrine markers (synaptophysin, chromogranin A and CD56) (Figure 3). Anti-VIP antibodies were not however available. Based on the pathological findings and the serum VIP level, we concluded to a recurrence of hepatic VIPoma. A mitotic count of 1 per 10 high power fields, and Ki-67 index estimated at 2%, categorized the tumor as a low-grade one. In an attempt to control the patient profuse diarrhea, she was started on a loading dose of short-acting octreotide at 50 micrograms subcutaneously every 12 h along with high daily doses of loperamide. The octreotide dose was escalated by increments of 100 micrograms every 48 h to a maximum dose of 100 micrograms every 8 h. No improvement of diarrhea in terms of consistency and frequency was noted on this treatment. Seen the presence of four different lesions in three different segments and the proximity of two of them to the inferior vena cava, surgery was considered unsafe. Thus, we opted for transarterial chemoembolization (TACE) using lipiodol and doxorubicin. On the first session, the lesions in segment I and III were treated with excellent immediate results (Figure 4). The symptoms substantially improved and the number of stools per day dropped to 1 or 2. The VIP level measured three days after the procedure was 54 pmol/l. Six weeks later, the remaining nodules were treated in the second session of TACE. Abdominal CT scan performed after 4 weeks showed complete necrosis of the lesions (Figure 5). Twelve months later, the patient remains completely symptom free.

## Discussion

Neuroendocrine tumors (NETs) are a rare type of tumor, deriving from neuro-ectodermal cells [1]. They comprise approximately 1–2% of all gastrointestinal tumors. While the liver is the most common site for metastasis of NETs, primary hepatic neuroendocrine tumors (PHNETs) are an exceedingly rare entity [2]. Less than 150 cases have been reported in the literature, and they comprise approximately 0.3% of all NETs [2]. This scarcity of cases makes it difficult for clinicians to diagnose PHNET accurately before biopsy or surgical resection of the tumor [1]. The histogenesis of PHNET is very poorly understood. It has been speculated that these tumors might result from the proliferation of either heterotopic aberrant pancreatic tissue in the liver or endocrine cells of neuroectodermal origin, particularly those from the intrahepatic biliary epithelium [3]. PHNECs have been described as typically slow growing tumors occurring mainly in females aged from 40 to 50 years. The right liver lobe seems to be more commonly affected which was the case for our patient [4].

Unlike most PHNETs who are typically endocrinologically silent and diagnosed either incidentally or at advanced stages, VIPomas are mainly revealed by large-volume diarrhea due to VIP unregulated overproduction. VIP is a peptide hormone that has been studied in numerous organ systems including the nervous system where it is mainly produced, the gastrointestinal, respiratory, cardiovascular, and endocrine systems. VIP is however majorly localized in the myenteric and submucosal neurons and nerve terminals in the gastrointestinal tract where it mediates through its receptors a myriad of functions such as regulating gastric acid secretion, anion secretion, pancreatic enzyme release, vasodilation and intestinal and cellular motility [5]. VIP also exhibits glycogenolytic effect on the liver causing hyperglycemia in up to 50% of cases which probably explains the newly diagnosed diabetes mellitus noted in this case [6]. Although VIP plasma levels are by definition increased in VIPomas, they may remain within normal range between episodes of diarrhea [7]. Patients with VIPoma can also present with facial flushing, skin rash, bloating, indigestion, nausea, vomiting, and documented unintentional weight loss which was the case for our patient [8].

The diagnosis of primary hepatic VIPoma is based on three pillars with regard to watery diarrhea, elevated VIP serum level and imaging study. Although radiological imaging is crucial in determining the lesions number, locations, size and thus guiding the treatment strategy, ultrasound (US), CT, and MRI lack sensitivity when it comes to PHNETs imaging. Thus, distinguishing PHNET from metastatic hepatic N and more common hepatic tumors, chiefly hepatocellular carcinoma and cholangiocarcinoma remains very challenging. PHNET features may vary largely appearing solid or cystic with diffuse or well-circumscribed margins and possible presence of areas of necrosis. The enhancement patterns of the solid PHNET are reported to be similar to those of hepatic metastases of NETS of the pancreas and the GI tract showing hyper-enhancement in hepatic arterial phase and washout in portal venous phase which was the case for our patient. [9]. As primary hepatic VIPomas are extremely rare, the available data in literature on their radiological presentation is almost non-existent. Octreotide scintigraphy has been showed in different studies to be highly sensitive in determining NET sites. However, it failed to detect the hepatic lesions in this case. In fact, octreotide scintigraphy is based on the visualization of octreotide-binding somatostatin receptors. However, the number of these receptors in some NET can be very limited resulting in a lower affinity for octreotide. Another possible explanation for our patient normal octreotide scan is the hepatic location itself. Liver metastases of NET are reported to be challenging to visualize with octreotide scan as they may accumulate the same amount of tracer as the normal hepatic cells surrounding the lesions. Yet, we do not know if these observations can be extrapolated to PHNET in general and VIPomas in particular [10].

In addition to this case, we were able to identify only three other cases of primary hepatic VIPoma based on a PubMed and a Google Scholar search of the English literature. The radiological features of these cases along with the clinical and biological ones are summarized in Table 1.

**Table 1.** Published cases of primary hepatic VIPomas.

Study, county, year	Age (years), sex, medical history	Clinical presentation	Remarkable biological features	Lesions number	Radiological features	Treatment	Ref.
Ayub *et al.*, Saudi Arabia, 1992	35, Male	Watery diarrhea		Solitary	Non-available	Surgical resection	[11]
Shah *et al.*, USA, 2005	78 male Diabetes mellitus Seizure disorder	Acute colonic pseudo-obstruction One year history of watery diarrhea	Hypokalemia High VIP level = 86.7 pg/ml (nl <= 50).	Solitary	Somatostatin (receptor scintigraphy: small focus of radiotracer uptake in the liver.	Octreotide and selective hepatic artery embolization.	[12]
Haiqing *et al.*, China, 2015	50, Male Hepatocellular carcinoma treated with an orthotopic liver transplantation	Watery diarrhea starting two months after the liver transplantation	Hypokalemia High Chromogranin level: 344.9 ng/ml	Solitary	Repeated CT and MRI: detected no lesions. Intraoperative ultrasonography revealed a 1 × 1 cm in the right liver	Radiofrequency ablation.	[13]

Similarly to PHNET, the mainstay of treatment of HPV is surgery resection or partial hepatectomy. The resectability rate for PHNET was reported to be nearly 70% and surgery seems to be effective and safe even when major hepatectomy is performed [14]. The 10-year survival rate after resection for PHNET and the recurrence rate were reported to be 70% and 18%, respectively, in a Review article including 48 patients [15]. Surprisingly, the survive rate does not seem to be influenced by the surgery type nor by the disease extent [14]. Post-surgical long-term follow-up is highly recommended by most authors to firmly rule out extra-hepatic localization [1]. To date there are no treatment guidelines specific to non resectable PHNETs. Several palliative therapies have been used such as systemic chemotherapy with somatostatin analogs, doxorubicin, streptozotocin, 5-fluorouracil, and TACE. Somatostatin analogues have been the mainstay of antisecretory treatment, a recent Meta-analysis demonstrated that octreotide reduced diarrhea in 65% [6]. In cases of resistance to somatostatin analogues, some authors suggest adding glucocorticoids, loperamide and opiates [8]. Due to NET high sensitivity to ischemia, TACE seems to be effective for treating hepatic metastases of extrahepatic NETs in terms of symptoms control and radiological response with a reported success rate of 92% [16]. Nevertheless, the available data on its effect on PHNET is limited to case reports where the procedures are performed with different cytotoxic agents such as doxorubivin, streptozotocin and miriplatin and the short-term results are highly variable [17,18]. Consequently, no conclusions can be drawn out of the current literature concerning TACE both short- and long-term efficiency in PHNET.

## Conclusion

This case highlights that long-term follow-up for patients with hepatic VIPoma is mandatory as recurrence could occur several years after curative surgical treatment. In these patients, the immediate focus should be on rapidly controlling the diarrhea to restore losses, prevent further complications and improve the patient quality of life. While surgery remains the mainstay of VIPomas treatment, in unresectable cases, TACE seems to be a safe and efficient therapeutic option.

**Executive summary**

- VIPomas are insidious functional neuroendocrine tumors.
- Hepatic localization of hepatic VIPomas is exceedingly rare.
- The immediate focus in patients with VIPomas should be on controlling the diarrhea.
- Transarterial chemoembolization is a safe and efficient therapeutic option in hepatic VIPomas.
- Long-term follow-up for patients with hepatic VIPoma is mandatory.

## References

[1] Song JE, Kim BS, Lee CH. Primary hepatic neuroendocrine tumor: a case report and literature review. World J. Clin. Cases 4(8), 243 (2016). 27574614 10.12998/wjcc.v4.i8.243 PMC4983697

[2] DeLuzio MR, Barbieri AL, Israel G, Emre S. Two cases of primary hepatic neuroendocrine tumors and a review of the current literature. Ann. Hepatol. 16(4), 621–629 (2017). 28611270 10.5604/01.3001.0010.0313

[3] Maire F, Couvelard A, Vullierme MP, Kianmanesh R, O'Toole D, Hammel P Primary endocrine tumours of the liver. Br. J. Surg. 92(10), 1255–1260 (2005). 15988793 10.1002/bjs.5073

[4] Yang K, Cheng YS, Yang JJ, Jiang X, Guo JX. Primary hepatic neuroendocrine tumors: multi-modal imaging features with pathological correlations. Cancer Imaging. 17(1). 20 (2017). 28683830 10.1186/s40644-017-0120-x PMC5501439

[5] Iwasaki M, Akiba Y, Kaunitz JD. Recent advances in vasoactive intestinal peptide physiology and pathophysiology: focus on the gastrointestinal system. F1000Res. 8, F1000 Faculty Rev-1629 (2019). 10.12688/f1000research.18039.1 PMC6743256 31559013

[6] Stueven AK, Kayser A, Wetz C, Amthauer H, Wree A, Tacke F Somatostatin analogues in the treatment of neuroendocrine tumors: past, present and future. Int. J. Mol. Sci. 20(12), (2019). 10.3390/ijms20123049 PMC6627451 31234481

[7] Grozinsky-Glasberg S, Mazeh H, Gross DJ. Clinical features of pancreatic neuroendocrine tumors. J. Hepato-Biliary-Pancreat Sci. 22(8), 578–585 (2015). 10.1002/jhbp.226 25689919

[8] Schizas D, Mastoraki A, Bagias G, Patras R, Lazaridis II, Arkadopoulos N Clinicopathological data and treatment modalities for pan-creatic vipomas: a systematic review. J. BUON 24(2), 415–423 (2019). 31127985

[9] Kellock T, Tuong B, Harris AC, Yoshida E. Diagnostic imaging of primary hepatic neuroendocrine tumors: a case and discussion of the literature. Case Rep. Radiol. 2014, 1–5 (2014). 10.1155/2014/156491 PMC4166617 25258691

[10] Critchley M. Octreotide scanning for carcinoid tumours. Postgrad Med. J. 73(861), 399–402 (1997). 9338023 10.1136/pgmj.73.861.399 PMC2431390

[11] Ayub A, Zafar M, Abdulkareem A, Ali MA, Lingawi T, Harbi A. Primary hepatic vipoma. Am. J. Gastroenterol. 88(6), 958–961 (1993). 8389095

[12] Shah KM, Ma X. Primary hepatic vipoma presenting as a colonic pseudo-obstruction: 469. Off. J. Am. Coll. Gastroenterol. ACG 100, S178 (2005).

[13] Haiqing W, Jiayin Y, Jian Y, Lunan Y. Intractable and dramatic diarrhea in liver transplantation recipient with vasoactive intestinal peptide-producing tumor after split liver transplantation: a case report. Transplant. Proc. 47(1), 171–173 (2015). 25596962 10.1016/j.transproceed.2014.07.078

[14] Quartey. Primary hepatic neuroendocrine tumor: what do we know now? World J. Oncol. 2(5), 209–216 (2011). 29147250 10.4021/wjon341w PMC5649681

[15] Knox CD, Anderson CD, Lamps LW, Adkins RB, Pinson CW. Long-term survival after resection for primary hepatic carcinoid tumor. Ann. Surg. Oncol. 10(10), 1171–1175 (2003). 14654473 10.1245/aso.2003.04.533

[16] Kress O, Wagner HJ, Wied M, Klose KJ, Arnold R, Alfke H. Transarterial chemoembolization of advanced liver metastases of neuroendocrine tumors–a retrospective single-center analysis. Digestion 68(2-3), 94–101 (2003). 14593235 10.1159/000074522

[17] Li XS, Zhang MC, Qu YC, Zhang XQ, Pan F, Liu YX. Diagnostic imaging of primary hepatic neuroendocrine tumors and treatment with transarterial chemoembolization: analysis of 6 cases. Zhonghua Gan Zang Bing Za Zhi Zhonghua Ganzangbing Zazhi Chin. J. Hepatol. 26(4), 294–297 (2018). 10.3760/cma.j.issn.1007-3418.2018.04.012 PMC12769230 29996342

[18] Shi C, Zhao Q, Dai B, Xie F, Yang J. Primary hepatic neuroendocrine neoplasm: long-time surgical outcome and prognosis. Medicine (Baltimore) 97(31), e11764 (2018). 30075602 10.1097/MD.0000000000011764 PMC6081183

